# Magnetoresistive biosensors with on-chip pulsed excitation and magnetic correlated double sampling

**DOI:** 10.1038/s41598-018-34720-0

**Published:** 2018-11-07

**Authors:** Kyunglok Kim, Drew A. Hall, Chengyang Yao, Jung-Rok Lee, Chin C. Ooi, Daniel J. B. Bechstein, Yue Guo, Shan X. Wang

**Affiliations:** 1 Department of Electrical Engineering, Stanford University, Stanford, CA United States; 2 Department of Electrical and Computer Engineering, University of California San Diego, La Jolla, CA United States; 3 Division of Mechanical and Biomedical Engineering, Ewha Womans University, Seoul, South Korea; 4 Department of Chemical Engineering, Stanford University, Stanford, CA United States; 5 Department of Mechanical Engineering, Stanford University, Stanford, CA United States; 6 Department of Materials Science and Engineering, Stanford University, Stanford, CA United States

## Abstract

Giant magnetoresistive (GMR) sensors have been shown to be among the most sensitive biosensors reported. While high-density and scalable sensor arrays are desirable for achieving multiplex detection, scalability remains challenging because of long data acquisition time using conventional readout methods. In this paper, we present a scalable magnetoresistive biosensor array with an on-chip magnetic field generator and a high-speed data acquisition method. The on-chip field generators enable magnetic correlated double sampling (MCDS) and global chopper stabilization to suppress 1/*f* noise and offset. A measurement with the proposed system takes only 20 ms, approximately 50× faster than conventional frequency domain analysis. A corresponding time domain temperature correction technique is also presented and shown to be able to remove temperature dependence from the measured signal without extra measurements or reference sensors. Measurements demonstrate detection of magnetic nanoparticles (MNPs) at a signal level as low as 6.92 ppm. The small form factor enables the proposed platform to be portable as well as having high sensitivity and rapid readout, desirable features for next generation diagnostic systems, especially in point-of-care (POC) settings.

## Introduction

The advent of biomedical technologies has brought an increased interest in discovering new methods to identify diseases earlier where treatments are often more effective, healthcare costs are lower, and patient outcomes are generally better. Over the past few decades, substantial effort has been made to identify new prognostic biomarkers^1–3^ and improve measurement sensitivity^4–7^. Magnetic biosensors have been studied extensively^8–12^, and giant magnetoresistive (GMR) biosensors in particular, have drawn considerable research interest because of their high transduction efficiency and the simple fact that biological samples are rarely magnetic, thus ensuring a low background signal, resulting in an extremely low, femto-Molar limit of detection^13^. Furthermore, GMR biosensors are matrix-insensitive^13^, capable of multiplexing^14,15^, and can be made temperature-insensitive^16^. With an increasing amount of research dedicated towards extending such biosensor applications, including the detection of DNA^17–21^, cancer biomarkers^13^, and cardiovascular biomarkers^15^, there is also a growing need for scalable, high-density biosensor arrays. Such arrays allow for simultaneous detection of many biomarkers from a single biological sample, enabling personalized healthcare, while saving time and reagent cost. Ideally, these high-density biosensor arrays would have a short readout time to enable kinetic monitoring, high sensitivity, and low power consumption. Yet the desire for large array size and short readout time are often at odds with each other. Most prior work has compromised the short readout time in favor of large array size.

The scalability of current magnetoresistive biosensors is primarily limited by its data acquisition speed. Conventional design methodology utilizes spectral analysis to measure minute signal changes, essentially lock-in detection with a narrow acquisition bandwidth^15,22–26^. This has been shown to accommodate arrays of up to 256 sensors^24^, but it suffers from long recording time (500 ms per readout channel) and requires even longer processing time. This long data acquisition time results in a significant time penalty for reading out the entire array (usually a few seconds per scan) and hinders the ability to monitor real-time reaction kinetics. Techniques such as multi-carrier excitation have been shown to reduce the readout time but are limited by the increased dynamic range and are sensitive to distortion in the readout electronics^27–29^. A recent study showed that time-domain analysis with magnetic correlated double sampling (MCDS) can be effectively utilized to measure signals from the sensor arrays^30^. However, the usage of an external Helmholtz coil and power amplifier requires a significant power consumption as well as a large form factor.

**Figure 1:**
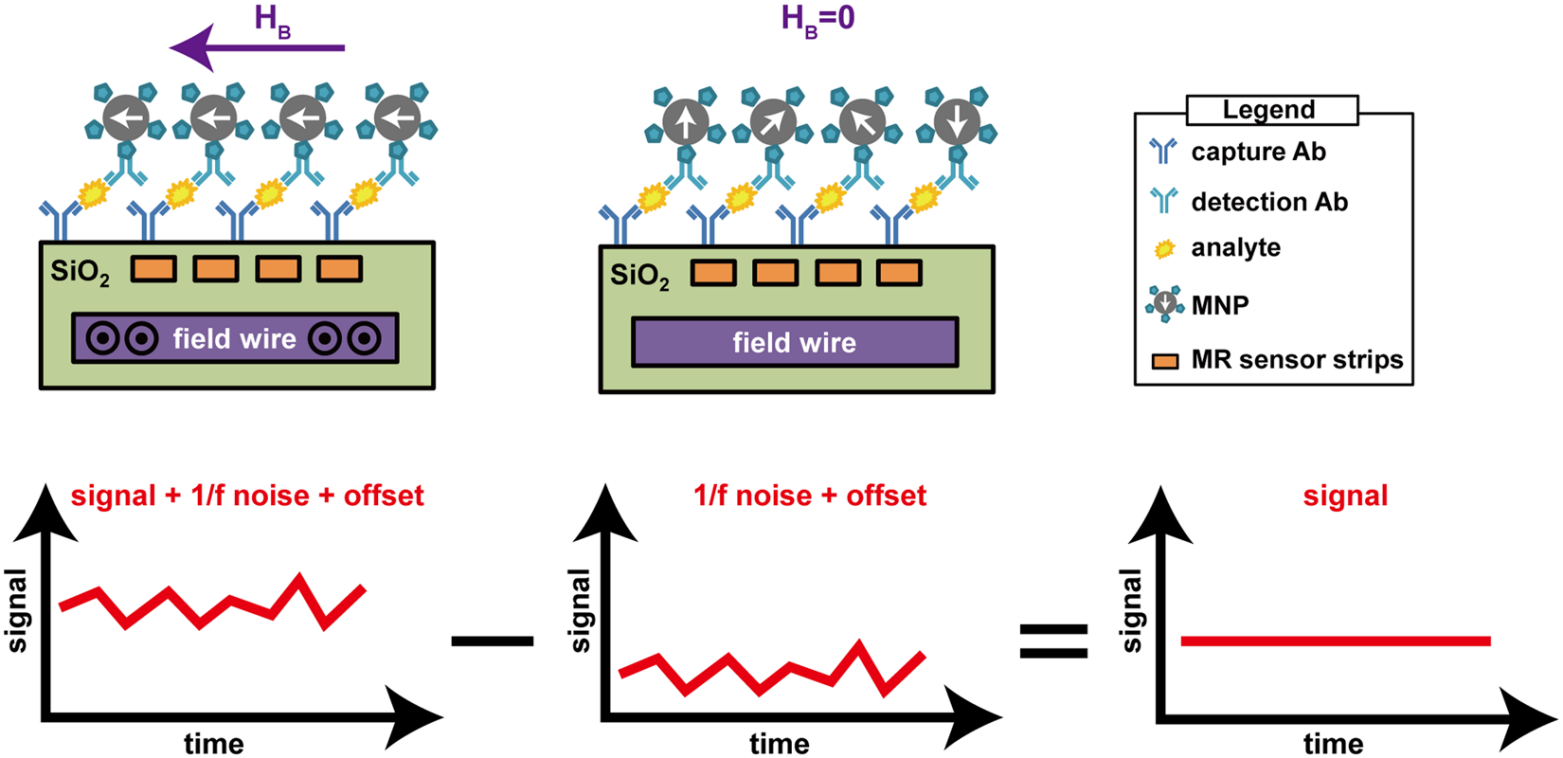
Overview of the proposed magnetic correlated double sampling technique with a GMR biosensor. A set of capture antibody, analyte of interest, and detection antibody form a sandwich structure tethering MNPs to the surface. When current is passed through the field wire, an excitation field is generated (left), the output voltage is a superposition of the signal, 1/f noise, and offset. When there is no excitation (middle), the output voltage measures only the 1/f noise and the offset. If the time difference between the two measurements is sufficiently small, 1/f noise and offsets are highly correlated so the subtraction of the two contains only the signal of interest (right).

In this work, we present a scalable magnetoresistive biosensor array with on-chip pulsed excitation and a readout circuit employing the MCDS technique, as illustrated in Fig. 1. The system consists of a GMR spin-valve (SV) sensor array with field generating strip line inductors embedded on the same chip directly under the sensors, and a custom analog front-end (AFE). The on-chip strip lines eliminate the need for a bulky external coil and its corresponding power amplifier while also making this design much more scalable. The on-chip field generators magnetize the magnetic nanoparticles (MNPs) tethered to the surface by a sandwich immunoassay (capture antibody, analyte of interest, and detection antibody conjugated to an MNP). The captured MNPs induce a local magnetic field that is sensed by the underlying magnetoresistance (MR) sensor. This MR change is detected using custom designed electronics with the MCDS technique to suppress the flicker (1/*f*) noise and offset, thus allowing one to extract the minute signal of interest in the time domain and reducing the readout time while preserving the same level of sensitivity. To perform MCDS, an excitation field generated by a strip line inductor implanted under the sensors is pulsed biphasically and the sensor resistance is measured twice within a very short period of time. The first measurement is taken with excitation field on, and the second, off. The first measurement contains the signal of interest (ΔMR) along with the 1/*f* noise and offset of the sensor and readout electronics, while the second measurement has only the 1/*f* noise and offset. By subtracting the second measurement from the first, the correlated noise and offset are eliminated, leaving only the MR change induced by the captured MNPs. In a biological assay, the temperature can change significantly, and usually induces signal artifacts that can be orders of magnitude larger than the signal of interest. A temperature correction technique is proposed to remove this dependence without using extra sensors or redundant measurements. Binding curves obtained from a magnetic assay demonstrate a minimum detectable signal of 6.92 ppm (~3,900 MNPs) and strong linearity in 20 ms.

## Results

### Magnetic biochips

**Figure 2:**
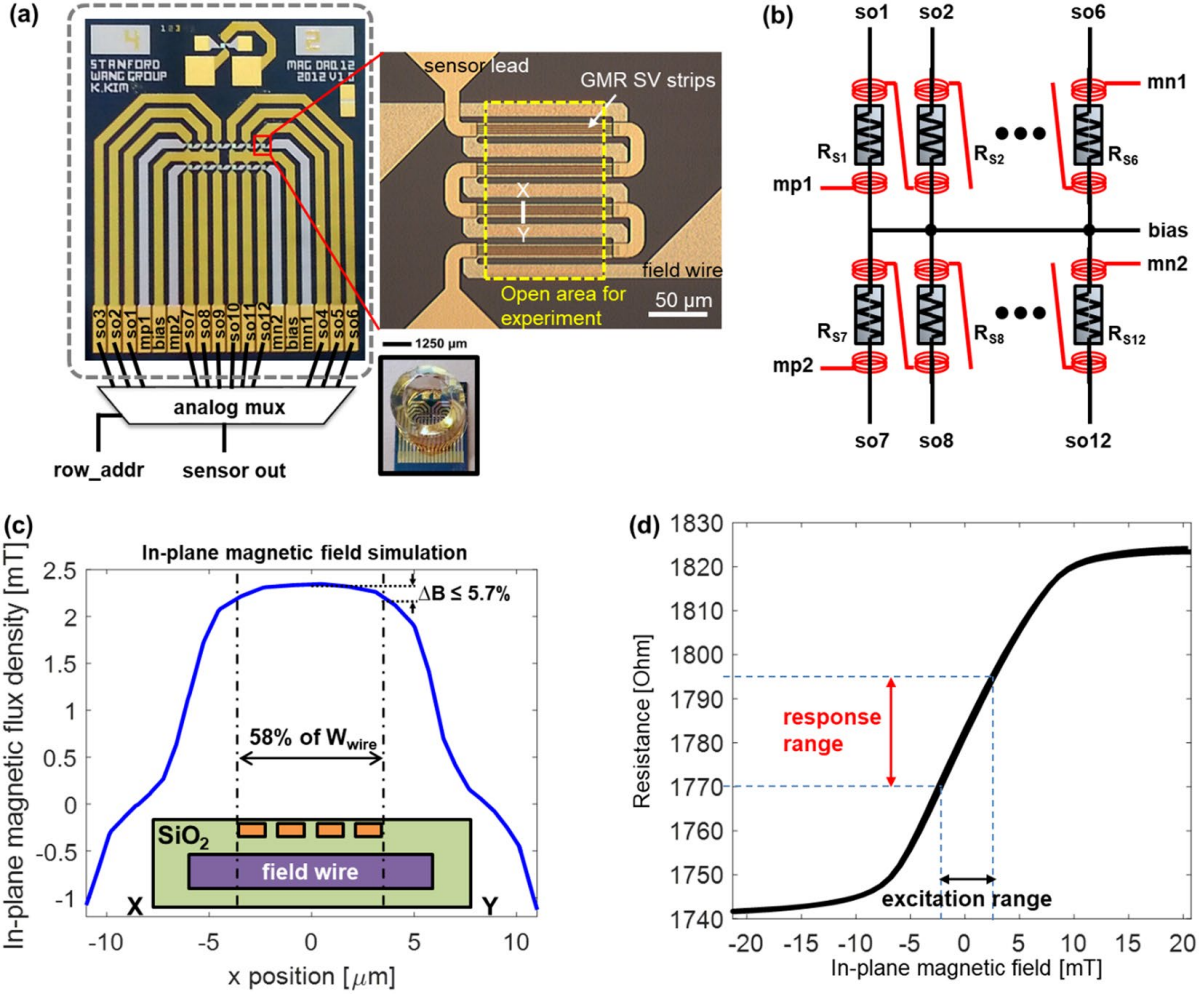
(**a**) Photograph of the fabricated biochip showing the detail of each sensor and image of the chip with a reaction well attached. Dashed rectangle shows the active area for experiments. (**b**) Connection of integrated strip line inductors to realize the MCDS technique. (**c**) Finite element simulation results for in-plane magnetic field generated by integrated strip line inductors across sensor strips. (**d**) Measured resistance versus in-plane magnetic flux density.

A 12-sensor GMR SV biochip with integrated magnetic field generators was designed and fabricated (Fig. 2). The sensors had a measured resistance of 1.78 kΩ ± 5.28 Ω with no magnetic field applied. The sensor resistance was designed by combining multiple stripes of GMR SV sensors in a parallel and series configuration to yield a moderate ~1.8 kΩ resistance with a large surface area (14,800 µm^2^) to increase the dynamic range. The measured MR ratio of the film was 7% after deposition but degraded to 5% due to stress induced during patterning and subsequent processing. The sensors had a sensitivity of 4.92 kΩ/T and most linear in ±2.5 mT range.

To detect superparamagnetic MNPs tethered to the surface of the sensor, a magnetic field is required to magnetize them^31^. This excitation field can be provided by either an external coil or an on-chip magnetic field generator. While external coils have several advantages, namely homogeneous fields and minimal sample heating, they suffer from a limited operating frequency range due to their large inductance, require substantially higher power, and have much larger size limiting their portability. Recent work has shown that it is able to overcome the switching speed limitation using a modified power amplifier^30^, but the power consumption and size are still significant. In this work, strip line inductors were fabricated below each of the sensors enabling high frequency operation (which is needed for the proposed MCDS technique) and small form factor with significantly reduced power consumption.

Several on-chip magnetic field generators have been previously reported^32–36^, but they suffered from poor field uniformity. To overcome this limitation, we propose a design where the sensors are localized within 58% of the strip line width at the center (Fig. 2c). The distance between the sensors and the strip lines was engineered by trading off the effective in-plane magnetic field and dielectric strength against the dielectric breakdown^37,38^. The resulting field generators produce an in-plane magnetic field of up to 2.3 mT from a 50 mA current, achieving 94% field uniformity across the sensor and a current-to-flux density conversion ratio of 46 μT/mA. These values are confirmed from both the finite element simulation and measurement data. The magnetic field density is measured at 50 nm above the GMR sensor surface. This vertical distance is chosen to represent the vertical distance of the MNPs in a practical immunoassay. It is noted that magnetic flux density decays reciprocally along the vertical direction. However the distance between the captured MNPs and the sensor surface is usually too small that such variations only cause minimal differences on the magnetic flux density. A finite element simulation showing magnetic flux density around a field wire and sensor strips is shown in Fig. S1(a). The calculated magnetic flux density showed only 1.4% change when the vertical height increased from 0 nm to 100 nm (Fig. S1(b)). To measure the resistance change due to the excitation field, the in-plane magnetic field was swept from −20 mT to 20 mT. The measured resistance curve shows the GMR effect saturates when the in-plane magnetic field is below −6 mT or above 8 mT (Fig. 2d). In this work, the ±2.5 mT region is utilized where the curve is mostly linear.

### Magnetic correlated double sampling and global chopper stabilization

Hybridization and binding assays generally take fifteen or more minutes to reach equilibrium^14,23,29^. As such, this measurement is long enough for 1/*f* noise (from both the sensors and the readout electronics) to severely distort the measurements. This is particularly true for GMR sensors which are known to have high 1/*f* noise due to their multi-layer structure with multiple interface layers^21,32,39,40^. Correlated double sampling (CDS) is a classical circuit technique to suppress 1/*f* noise^41,42^. Briefly, two sequential measurements are made where one contains the signal of interest with noise added, and the other, only the noise. By subtracting these two measurements, the correlated noise is removed at the expense of doubling the uncorrelated (white) noise. To apply this technique to magnetic sensors, one can take a measurement with the magnetic field turned on (a sum of the signal, *V*_MR_, the non-magnetic portion of the sensor, *V*_BASE_, and the noise) and another measurement with the magnetic field off (only *V*_BASE_ and the noise). Provided that the temporal difference between the two measurements (Δ*t*) is sufficiently small, the 1/*f* noise is highly correlated and thereby eliminated by subtraction. This technique has been referred to as MCDS^30^.

Ideally only two samples are required; however, the data is still prone to having residual noise. To achieve the best 1/*f* noise suppression, conventional CDS circuits are implemented on an integrated circuit, where designers can utilize the symmetry and close proximity in layout between the signal path and the reference path to minimize process variations. However, for a prototype design presented in this work using off-the-shelf components, there are higher residual 1/*f* noise and offset voltages after MCDS, and the following circuits can contribute additional noise and offsets as well. To overcome this, we have extended the MCDS technique to a four-phase operation with global chopper stabilization where measurements are made with a positive magnetic field (*H*_B_ > 0), no magnetic field (*H*_B_ = 0), a negative magnetic field (*H*_B_ < 0), and again with no magnetic field applied (*H*_B_ = 0). The measured magnetoresistance components are thus ∆*MR*, 0, -∆*MR*, and 0, respectively.

**Figure 3:**
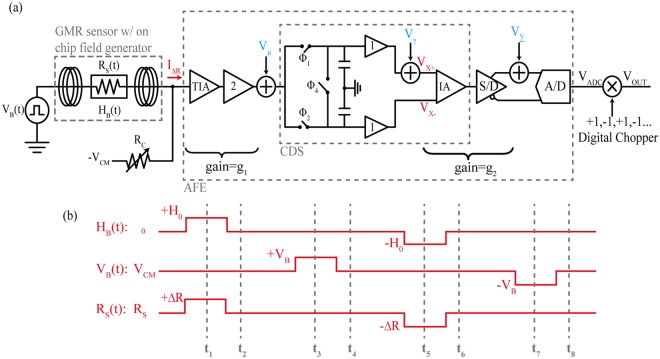
(**a**) Simplified schematic of the analog front-end. *V*_μ_*, V*_*ɣ*_ and *V*_*ɛ*_ are the noise and offset errors to be removed. (**b**) Timing diagram and resistance changes responding to magnetic field *H*_B_ and bias voltage *V*_B_.

A prototype readout analog front-end (AFE) was designed using off-the-shelf components and assembled in house on custom printed circuit boards (PCBs). An overview of the signal path is shown in Fig. 3(a) and a detailed schematic of the signal path and associated timing needed to implement the MCDS operation is shown in Fig. S2. To measure the 1/*f* noise and offset-free signal, the magnetic field, *H*_B_ is pulsed at 2.3 mT for 40 µs. This excites the magnetoresistance of the sensor, but the bias voltage, *V*_B_ is kept constant at common-mode voltage (1.65 V). The MCDS operation (*t*_1_ and *t*_2_ in Fig. 3b) is performed in the analog domain to subtract measurements with *H*_B_ on and off, thus resulting in a measurement of the magnetoresistance, *V*_MR_:

1 $${V}_{{\rm{M}}{\rm{R}}}=(\mathop{\underbrace{(\frac{{V}_{{\rm{C}}{\rm{M}}}}{{R}_{{\rm{S}}}+{\rm{\Delta }}R}+\frac{-{V}_{{\rm{C}}{\rm{M}}}}{{R}_{{\rm{C}}}})}}\limits_{{\bf{F}}{\bf{i}}{\bf{r}}{\bf{s}}{\bf{t}}\,{\bf{m}}{\bf{e}}{\bf{a}}{\bf{s}}.{\boldsymbol{,}}\,{{\bf{H}}}_{{\bf{B}}}{\boldsymbol{ > }}0}-\mathop{\underbrace{(\frac{{V}_{{\rm{C}}{\rm{M}}}}{{R}_{{\rm{S}}}}+\frac{-{V}_{{\rm{C}}{\rm{M}}}}{{R}_{{\rm{C}}}})}}\limits_{{\bf{S}}{\bf{e}}{\bf{c}}{\bf{o}}{\bf{n}}{\bf{d}}\,{\bf{m}}{\bf{e}}{\bf{a}}{\bf{s}}.,\,{{\bf{H}}}_{{\bf{B}}}{\boldsymbol{=}}0}){G}_{{\rm{P}}{\rm{A}}{\rm{T}}{\rm{H}}}\approx -\frac{{\rm{\Delta }}R}{{R}_{{\rm{S}}}^{2}}{G}_{{\rm{P}}{\rm{A}}{\rm{T}}{\rm{H}}}{V}_{{\rm{C}}{\rm{M}}}$$

where Δ*R* is the change in resistance due to the applied magnetic field, *V*_CM_ is the common-mode voltage, *R*_S_ is the sensor resistance without an applied magnetic field (~1.8 kΩ), *R*_C_ is the resistance of the bleed resistor (~1.7 kΩ), and *G*_PATH_ is the transimpedance gain of the entire signal path (~112.04 dBΩ). This expression is simplified by taking the Taylor expansion and discarding all the 2^nd^ and higher order terms.

To measure the base resistance, the bias voltage, *V*_B_ is pulsed at 5 mV for 40 µs, resulting in (*t*_3_ and *t*_4_ in Fig. 3b):

2 $${V}_{{\rm{BASE}}}=(\mathop{\underbrace{(\frac{{V}_{{\rm{CM}}}+{V}_{{\rm{B}}}}{{R}_{{\rm{S}}}}+\frac{-{V}_{{\rm{CM}}}}{{R}_{{\rm{C}}}})}}\limits_{{\bf{First}}\,{\bf{meas}}{\boldsymbol{.}}}-\mathop{\underbrace{(\frac{{V}_{{\rm{CM}}}}{{R}_{{\rm{S}}}}+\frac{-{V}_{{\rm{CM}}}}{{R}_{{\rm{C}}}})}}\limits_{\mathrm{Second}\,\,{\bf{meas}}{\boldsymbol{.}}}){G}_{{\rm{PATH}}}=\frac{{V}_{{\rm{B}}}}{{R}_{{\rm{S}}}}{G}_{{\rm{PATH}}}$$

Thus, allowing *R*_S_ and Δ*R* to be readily calculated from the acquired data.

To implement the global chopper stabilization, the procedure above is repeated (*t*_5_ to *t*_8_ in Fig. 3b), but with the opposite polarities of *V*_*B*_ and *H*_B_. The procedure is shown in Fig. 3b and summarized in Table S1. The noise is removed by averaging the digitized output voltage at *t*_4_ and *t*_8_. One data point requires four sets of MCDS operations, each having four phases of 40 µs. The total time to record one data point is 640 µs, resulting in a field frequency of 1.5625 kHz.

The non-magnetoresistive component is coherently cancelled by the baseline suppression circuit to improve the dynamic range of the signal path and is not seen by the CDS circuit. Other circuit non-idealities such as residual 1/*f* noise and offset from the instrumentation amplifier, offset from the MCDS circuit caused by charge injection, and mismatch in the switches are suppressed by global chopper stabilization. The sample and hold and differencing operations are performed by the MCDS circuit prior to digitization, modulating the signal in the analog domain; while the demodulation from the bipolar magnetic field is done in the digital domain. The low frequency non-idealities are up-modulated to the chopping frequency and thus filtered out digitally. The penalty for the additional white noise is alleviated by low pass filtering and averaging of the incoming data. This MCDS technique with the global chopper stabilization is particularly powerful and only requires that the magnetic field driver can operate in a bipolar fashion.

**Figure 4:**
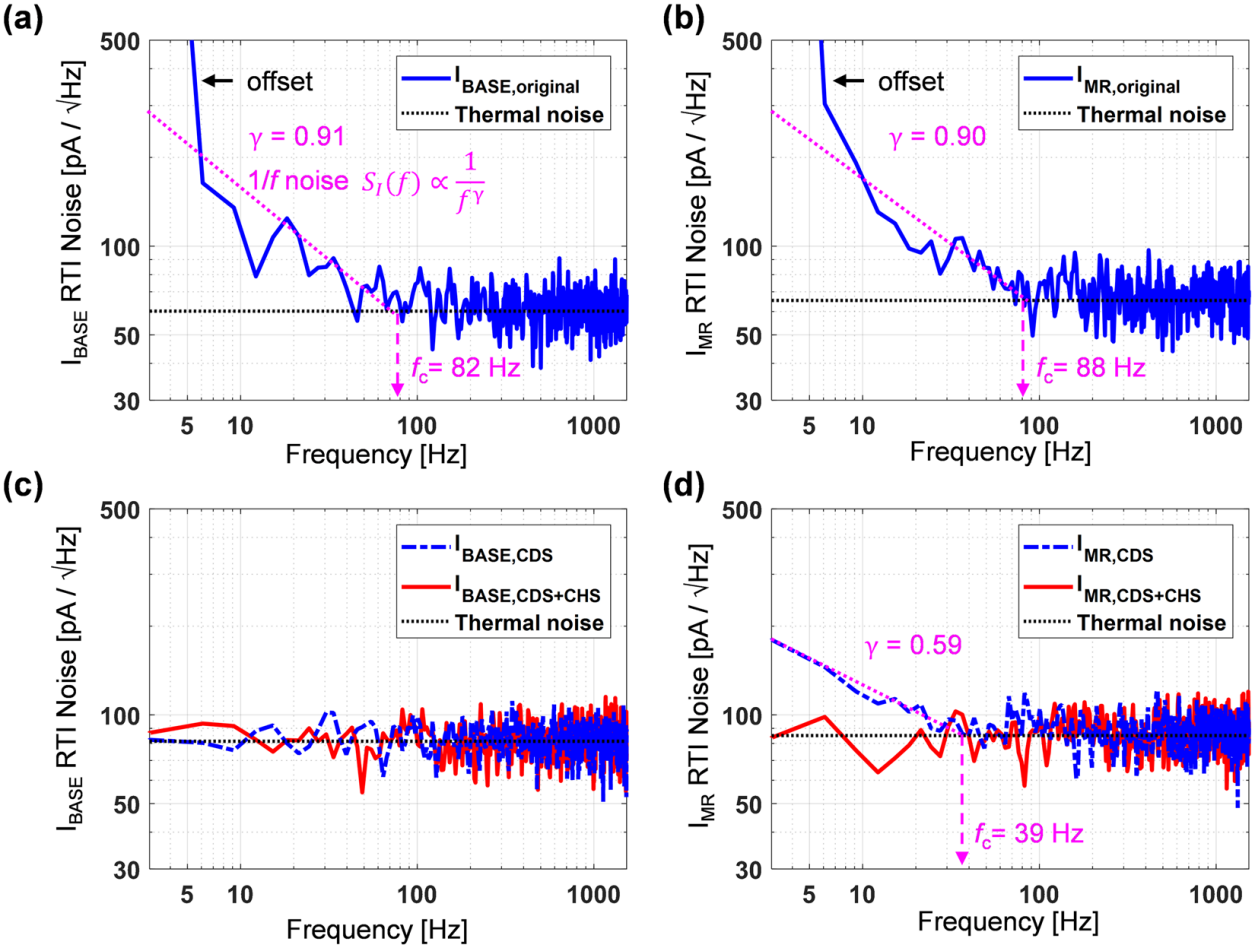
Effect of magnetic correlated double sampling and global chopper stabilization techniques. (**a**,**b**) Measured input-referred current noise without the proposed techniques. (**c**,**d**) Measured data showing the removal of the 1/*f* noise by magnetic correlated double sampling and global chopper stabilization for the resistive and Magnetoresistive components, respectively.

Noise data are measured both with and without the proposed MCDS and chopper stabilization. Figure 4 illustrates the power of this technique to suppress 1/*f* noise. Without these techniques, the sensor and AFE have significant energy at low frequencies that is reciprocally proportional to *f*^*ɣ*^ where *ɣ* is 0.91 for the nominal resistance and 0.90 for the magnetoresistance (Fig. 4a,b). The 1/*f* noise corner frequency (where the flicker noise intercepts the white noise floor), *f*_c_, are 82 Hz and 88 Hz for the resistive and magnetoresitive components, respectively. Using the proposed technique, both the sensor resistance (Fig. 4c) and magnetoresistance (Fig. 4d) see significantly lower noise contribution in this frequency range. Applying only the MCDS results in the resistive 1/*f* noise being entirely removed, but the magnetoresistive component still has residual 1/*f* noise (*ɣ* = 0.59 and *f*_c_ = 39 *Hz)*. This residual 1/*f* noise comes from the gain of *V*_MR_ being three decades smaller than that of *V*_BASE_. Therefore the 1/*f* noise of IA, S/D, and ADC are non-negligible in *I*_MR,CDS_. This residual 1/*f* noise is further suppressed by the global chopper stabilization as illustrated in Fig. 4d.

### Temperature correction

An MR sensor may experience a rapid temperature change in certain applications such as bioanalyte detections because the experimental procedure introduces cold reagents directly on to the surface of the MR sensors. For example, MNPs are conveyed through cold phosphate buffer solution^14^ or hexane^43,44^. When the solution containing MNPs is applied to the sensor, it experiences a drastic temperature drop because of the temperature difference between its surface and the solution. The local heat from the on-chip field generators due to the non-zero resistance can also influence on the signal degradation of the MR sensor. Without proper treatments to desensitize temperature effect on MR sensors, it is hard to distinguish the signal change due to the magnetic field change from the signal variation by temperature fluctuation in measurements^16^.

Accounting for temperature perturbation, *V*_BASE_ and *V*_MR_ from Eqs (1) and (2) become

3 $${V}_{{\rm{MR}},{\rm{measured}}}(t)=-\frac{{G}_{PATH}\,{V}_{{\rm{CM}}}\,{\rm{\Delta }}{R}_{0}(1+{\rm{\beta }}{\rm{\Delta }}T\,)}{{({R}_{{\rm{S}}0}(1+{\rm{\alpha }}{\rm{\Delta }}T))}^{2}}=-\frac{{G}_{{\rm{PATH}}}{V}_{{\rm{CM}}}{\rm{\Delta }}{R}_{0}}{{R}_{{\rm{S}}0}^{2}}\cdot \frac{1+{\rm{\beta }}{\rm{\Delta }}T\,}{{(1+{\rm{\alpha }}{\rm{\Delta }}T)}^{2}}$$

4 $${V}_{{\rm{BASE}},{\rm{measured}}}(t)=-\frac{{V}_{B}}{{R}_{{\rm{S}}0}(1+{\rm{\alpha }}{\rm{\Delta }}T)}{G}_{{\rm{PATH}}}$$

where *R*_S0_(1 + α∆*T*) is substituted for *R*_S_, and ∆*R*_0_(1 + β∆*T*) for ∆*R*. *R*_S0_ and Δ*R*_0_ are the nominal resistance and magnetoresistance at *t* = 0; α and β are the temperature coefficients (TCs) of the resistance (*R*_S_) and magnetoresistance (∆*R*). The temperature dependence (i.e., *α∆T* and β∆*T*) can be extracted from two sets of measurements, namely, *V*_BASE,measured_ and *V*_MR,measured_ at time 0 and *t*. After normalizing the signals at time *t* to their initial magnitude at time 0 and rearranging terms,

5 $${\rm{\alpha }}{\rm{\Delta }}T=\frac{{V}_{{\rm{BASE}},{\rm{measured}}}(0)}{{V}_{{\rm{BASE}},{\rm{measured}}}(t)}-1$$

6 $${\rm{\beta }}{\rm{\Delta }}T=\frac{{V}_{{\rm{MR}},{\rm{measured}}}(t)}{{V}_{{\rm{MR}},{\rm{measured}}}(0)}\cdot {(1+{\rm{\alpha }}{\rm{\Delta }}T)}^{2}-1=\frac{{V}_{{\rm{MR}},{\rm{measured}}}(t)}{{V}_{{\rm{MR}},{\rm{measured}}}(0)}\cdot {(\frac{{V}_{{\rm{BASE}},{\rm{measured}}}(0)}{{V}_{{\rm{BASE}},{\rm{measured}}}(t)})}^{2}-1.$$

The correction factor is defined as the reciprocal of the temperature dependent factors in Eq. (3).

7 $$CF\triangleq \frac{{(1+{\rm{\alpha }}{\rm{\Delta }}T)}^{2}}{1+{\rm{\beta }}{\rm{\Delta }}T}$$

Finally, the temperature dependent term can be eliminated by multiplying the *CF* with the original measurement where

8 $${V}_{{\rm{MR}},{\rm{corrected}}}(t)={V}_{{\rm{MR}},{\rm{measured}}}(t)\cdot CF=-\frac{{G}_{{\rm{PATH}}}{V}_{{\rm{CM}}}{\rm{\Delta }}{R}_{0}}{{R}_{{\rm{S}}0}^{2}}.$$

**Figure 5:**
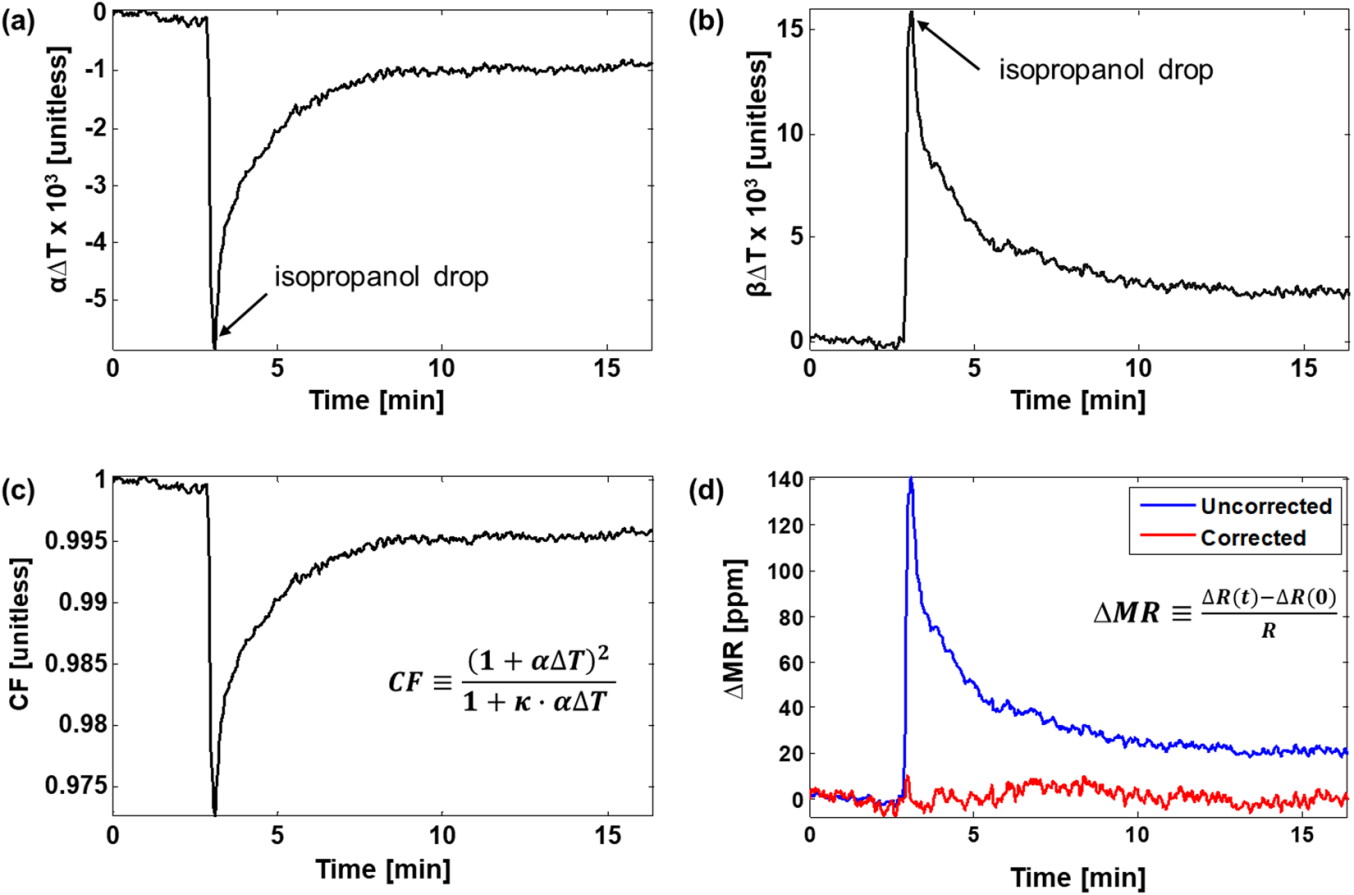
Temperature desensitization using the proposed temperature correction scheme. (**a**,**b**) Isopropanol alcohol whose temperature is 10 °C creates an abrupt change in the α∆*T* (resistive) and β∆*T* (magnetoresistive) curves when added at *t* = 3 minutes (**c**) CF curve derived using Eq. (7). (**d**) Sensor data measured before and after correction showing the removal of the temperature induced signal.

To characterize the temperature coefficients (α and β), a one-time procedure where a sudden temperature change is induced by dropping low temperature (10 °C) isopropanol solution onto the sensor surface (20 °C). Figure 5a,b show the measured α*∆T* and β*∆T* curves derived by applying *V*_BASE,measured_ and *V*_MR,measured_ to Eqs (5) and (6), respectively. From that, the CF curve is obtained (Fig. 5c), that can then be used to desensitize the MR change from the temperature change. A temperature desensitized result after applying temperature correction (Eq. (8).) to *V*_MR,measured_ is shown in Fig. 5d in comparison with the uncorrected signal. The signal is translated to ∆*MR* in ppm, the ratio of the *∆R* change to *R*, to normalize out sensor process variation. Compared with the raw *∆MR* before correction, the temperature corrected *∆MR* is stable regardless of temperature fluctuation induced by low temperature isopropanol. The standard deviation of corrected *∆MR* is 3.29 ppm when 64 consecutively measured samples are averaged and plotted as a single data point, or 20 ms per measurement. This translates to a minimum signal level of 6.58 ppm (SNR > 6 dB) or a 2.4 μT field change. Without temperature correction, the raw signal suffers from a large fluctuation of 140 ppm (equivalent to 50.2 μT) and the *∆R* signal corresponding to the magnetic field change cannot be distinguished from that to the temperature variation. The noise performance can be improved through further averaging, at the expense of longer readout time. Noise and sampling time data are plotted in Fig. S3 to illustrate this tradeoff. This exact same tradeoff also exists in the frequency domain measurement techniques where the lock-in bandwidth is inversely proportional to the length of the data^29^.

### Magnetic Assay

**Figure 6:**
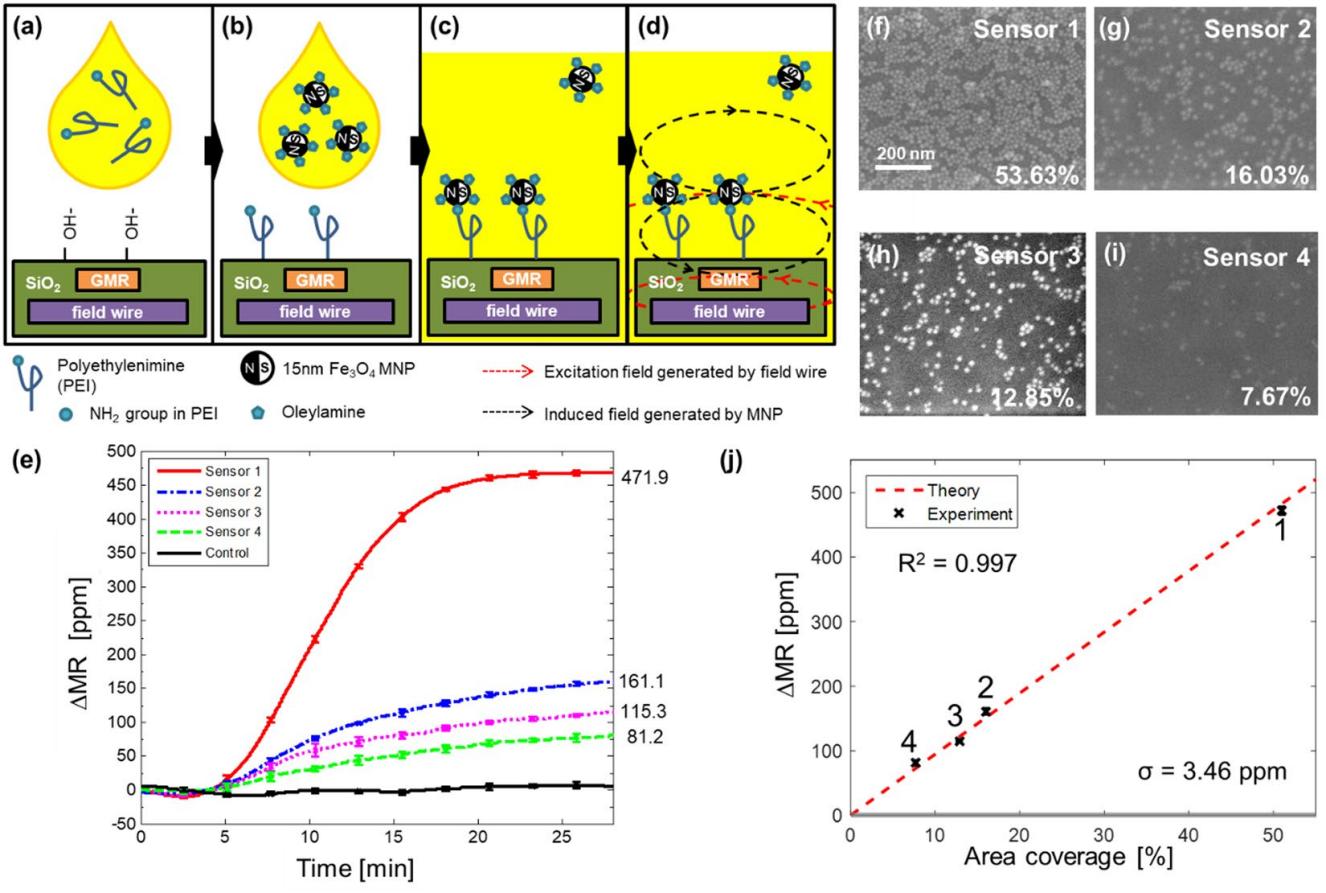
Quantitative detection of superparamagnetic nanoparticles in a proof-of-concept experiment. (**a**) The surface is functionalized using ozone plasma then PEI in chloroform solution is applied. (**b**) The residue is cleaned then MNPs in hexane solution are introduced. (**c**) MNPs are tightly bound to PEI through ligand exchange. (**d**) When integrated field wires generate magnetic excitation field around GMR sensors, MNPs in close proximity produce induced field that is sensed by the GMR sensors. (**e**) Measured binding curves of different PEI coverages. (**f**–**i**) Scanning electron microscopic images showing MNPs bound to sensor surface. Coverage area is measured using image processing software. (**j**) ∆MR versus area coverage by MNPs curve. ∆MR is proportional to area coverage, which corresponds to the number of MNPs. Error bars represent one standard deviation. Dashed line represents a theoretical calculation of ∆MR from 0 to 100% coverage.

We performed MNP measurements to simulate bioassays using the method previously reported^43^. It quantitatively characterizes the influence of superparamagnetic MNPs in close proximity on the fabricated GMR SV sensors. The chemical absorption of oleylamine to a polyethyleneimine (PEI) functionalized surface is used in this experiment (Fig. 6a–c). Oleylamine coated MNPs are replaced with a NH_2_ functional group in PEI by ligand exchange and a covalent bond forms between the MNPs and PEI. As such, the bound MNPs are located very close to GMR sensors. When the on-chip strip line inductors are turned on to generate a magnetic excitation field, the MNPs are magnetized and induce a field reducing the effect of the excitation field on the GMR sensors (Fig. 6d). The change in magnetoresistance is proportional to the number of bound MNPs and can be modulated by the surface coverage of PEI. Controlling the PEI coating time yields different coverages across the sensor array. Therefore, the PEI coverage area on the sensor surface simulates the detection of biological analytes at different concentrations using the sensors.

Measured binding curves are shown in Fig. 6e where the MNPs were added 5 minutes after starting the experiment. The sensors with PEI exhibit binding curves that saturate after 27 minutes whereas the three control sensors produce no signal because of the thick passivation. Sensors 1 to 4 had the highest to lowest PEI coverage, respectively (Fig. 6f–i). The MNP coverage percentiles were calculated by analysis of scanning electron microscopy (SEM) images and are labeled on each image. The ∆*MR* signals show strong linear trend with respect to the area coverage, as shown in Fig. 6j. From theoretical calculations^29^, it can be derived that the average MR change induced by a single MNP at 2.3 mT is 3.2 µΩ/MNP. Using this relationship and the geometries of the MNP and sensor, a theoretical linear relationship between the ∆*MR* signal versus area coverage is plotted along with the measured data (red dashed line in Fig. 6j). The measurements show strong agreement with the calculations. The standard deviation of the control data (3.46 ppm) determines the minimum detectable signal level is 6.92 ppm with 6 dB SNR, which is equivalent to 0.77% area coverage with 3,849 MNPs captured, or a change of 2.45 µT in magnetic flux density with a dynamic range of 47.2 dB.

It is worth noting that the vertical distance of an MNP-detection antibody-target analyte-capture antibody sandwich structure is longer than the MNP-PEI structure. Even though the change in magnetic flux density at these locations are negligible (Fig. S1(b)), the magnetic interaction between the nanoparticle and the GMR free layer may cause the signal level to fluctuate. Within the range of 0 to 100 nm, the net effect only causes a slight shift (6%) in the signal level^45^. Since the height of the captured MNP from a bioassay usually falls in this range, it is reasonable to extend the results from this section to a more generalized immunoassay application. The detection limit of MCDS approach is expected to be similar to that of our prior work^29^, but MCDS permits us to accommodate many more sensors on a single chip.

## Discussion

In summary, the GMR SV sensor array with on-chip field generators and the time-domain analysis method presented demonstrate great improvement in data acquisition speed, over 50× times faster than the conventional frequency-domain method. This enables the biosensor array to be highly scalable and extremely multiplexed. As a proof-of-concept for a large-scale biosensor array, a 12-sensor GMR SV biosensor array with integrated field generators was fabricated and characterized. The system has demonstrated a minimum detectable field change of 2.45 µT with a dynamic range of 42.7 dB. This result elucidates the proposed method can be readily applicable to build a large-scale GMR biosensor array system.

## Methods

### Fabrication of strip line inductors

Strip line inductors were fabricated by growing a thermal oxide (SiO_2_) insulating layer (1 μm thick) on a silicon wafer using an annealing furnace (Thermco Oxidation Furnace). Trenches (500 nm deep and 12 µm wide) were etched using a plasma etcher (Applied Materials P5000 Etcher). The remaining photoresist used for the lithography to define the trenches was intentionally not removed at this step. A metal film stack consisting of Ti (5 nm)/Au (490 nm)/Ti (5 nm) was deposited using an E-beam evaporator (Innotec ES26C E-Gun Evaporator) at 1 Å/sec to reduce the surface roughness. A lift-off was performed followed by removal of the remaining photoresist. Lastly, a global SiO_2_ passivation layer (500 nm) was deposited over the entire chip using PECVD (PlasmaTherm Shuttlecock PECVD System).

### Fabrication of GMR SV sensors

A blanket ion milling (MRC Reactive Ion Etcher) step was performed for 60 seconds to reduce surface roughness. A GMR SV film stack composed of a seed layer (4.5 nm)/Ir_0.2_Mn_0.8_ (8 nm)/Ni_0.8_Fe_0.2_ (3 nm)/Ru (0.9 nm)/Ni_0.8_Fe_0.2_ (3 nm)/Co_0.9_Fe_0.1_ (0.6 nm)/Cu (2.6 nm)/Co_0.9_Fe_0.1_ (0.8 nm)/Ni_0.8_Fe_0.2_ (6 nm)/Ru (0.9 nm)/Ta (5 nm) was deposited under a fixed directional magnetic field to set the initial magnetization (AJA UHV Deposition Chamber) and selectively patterned into stripes above the strip line inductors using ion milling (MRC Reactive Ion Etcher). The film stack thicknesses were selected based on Morais *et al*.^46^ and iteratively optimized. Resulting GMR SV stripes (125 μm long, 1 μm wide, and 1 μm spacing) were connected in parallel (n = 4) and series (n = 4) by Ti (5 nm)/Au (490 nm)/Ti (5 nm) by metal leads deposited using e-beam evaporation and lift-off. A total of 12 sensors were fabricated on each chip. A thin insulation layer consisting of SiO_2_ (15 nm)/Si_3_N_4_ (15 nm)/SiO_2_ (15 nm) was deposited using HDPCVD (PlasmaTherm Versaline HDP VCD System) while keeping the temperature below 200 °C to preserve the magnetization of the GMR sensors. Lastly, a thick SiO_2_ (150 nm)/Si_3_N_4_ (150 nm)/SiO_2_ (150 nm) passivation was deposited to protect the leads. The thick oxide was removed on all but three of the sensors that were subsequently used for negative controls. The entire fabrication process is shown in Fig. S4.

### Magnetic nanoparticle preparation

15 nm Fe_3_O_4_ monodisperse superparamagnetic nanoparticles (MNP) with an oleic acid coating were purchased from Ocean Nanotech (#SOR-15–50). The MNP were sonicated in chloroform, removed and mixed with oleylamine (O7805, Sigma Aldrich), and separated using a magnet. The collected MNP were re-suspended in hexane and stored at 4 °C for all future experiments.

### Magnetic sensor functionalization

The GMR sensor chip was placed in an UV ozone plasma chamber for 30 seconds to remove organics. The chip was then immersed in a chloroform solution containing 8% (by weight) PEI (408727, Sigma Aldrich) for 60 seconds. The chip was subsequently rinsed for 30 seconds in ethanol and air-dried.

### Magnetic assay

A fluidic reservoir was created on top of the GMR sensor array using a ¼” piece of Tygon tubing (57547, Tygon) adhered with two-part epoxy. The functionalized chip was connected to the readout electronics (described below) using an edge connector (ST80X-18S(50), Hirose Electric). After five minutes, MNPs were added while the sensors were continually readout to observe binding. All experiments were performed for 30 minutes to complete the reaction.

### Magnetic sensor characterization

GMR sensors were connected to a custom-built measurement station consisting of a Helmholtz coil driven by a power amplifier (BOP-20-20M, Kepco), a gaussmeter (420 Gaussmeter, Lakeshore), and a multimeter (HP34401A, Hewlett Packard). All instruments were controlled by a PC running LabVIEW to sweep the magnetic field while measuring the resistance and resulting magnetic field. Each data point was averaged (n = 100) to minimize the impact of noise.

### Analog front-end (AFE)

Custom readout electronics were designed using parts purchased from Digikey and assembled in house on custom printed circuit boards (PCBs) purchased from Advanced Circuits. The signal path consists of a transimpedance amplifier (AD8655, Analog Devices) with a programmable bleed resistor (implemented using an R-2R structure) to remove non-signal current^24^, a gain stage (AD8655, Analog Devices), and the proposed MCDS network built using switches (ADG712, Analog Devices), amplifiers (AD8656, Analog Devices), and an instrumentation amplifier (AD8422, Analog Devices). The single-ended output is converted to a differential signal (S/D) with a gain of 2 V/V (ADA4941-1, Analog Devices) to drive an analog-to-digital converter (AD7690, Analog Devices). An overview of the signal path is shown in Fig. 2(a). The strip line inductors are driven using a custom designed bipolar power amplifier (Fig. S5). The circuit consists of an amplifier (LT6202, Linear Technology) driving a PMOS transistor (IRLML2244, Infineon) connected to the strip line inductors. The strip line current is sensed using a shunt resistor and a differential amplifier (AD8211, Analog Devices), digitized (AD7942, Analog Devices), and then fed back to realize closed-loop control. The analog-to-digital converter (ADC) and switching logic is implemented on an FPGA (XC3S250E, Xilinx) and a microprocessor (NUC123, Nuvoton) is used to communicate to an external PC via USB. A block diagram of the proposed system is shown in Fig. S6 along with a photograph of the assembled PCB in Fig. S7.

## Electronic supplementary material

Supplementary material

## Data Availability

The datasets generated during and/or analysed during the current study are available from the corresponding author on reasonable request.
