# Supplementary material for: Magnetoresistive biosensors with on-chip pulsed excitation and magnetic correlated double sampling
Source: Sci Rep. 2018 Nov 7;8:16493. doi: 10.1038/s41598-018-34720-0 (PMC6220270; doi:10.1038/s41598-018-34720-0)
Supplement: Supplementary file 1 — Supplementary material [file 41598_2018_34720_MOESM1_ESM.docx]

**Supplementary Material**

**Title:** Magnetoresistive biosensors with on-chip pulsed excitation and magnetic correlated double sampling

**Author names and affiliation:**

Kyunglok Kim^a^, Drew A. Hall^b^, Chengyang Yao^a^, Jung-Rok Lee^c^, Chin C. Ooi^d^, Daniel J. B. Bechstein^e^, Yue Guo^a^, Shan X. Wang^a,f,*^

^a^ Department of Electrical Engineering, Stanford University, Stanford, CA, United States

^b^ Department of Electrical and Computer Engineering, University of California San Diego, CA, United States

^c^ Division of Mechanical and Biomedical Engineering, Ewha Womans University, Seoul, South Korea

^d^ Department of Chemical Engineering, Stanford University, Stanford, CA, United States

^e^ Department of Mechanical Engineering, Stanford University, Stanford, CA, United States

^f^ Department of Materials Science and Engineering, Stanford University, Stanford, CA, United States

**Corresponding author:** [sxwang@stanford.edu](mailto:sxwang@stanford.edu)

**Present Address:**

Stanford University

McCullough Building, Rm. 351

Stanford, CA 94305-4045


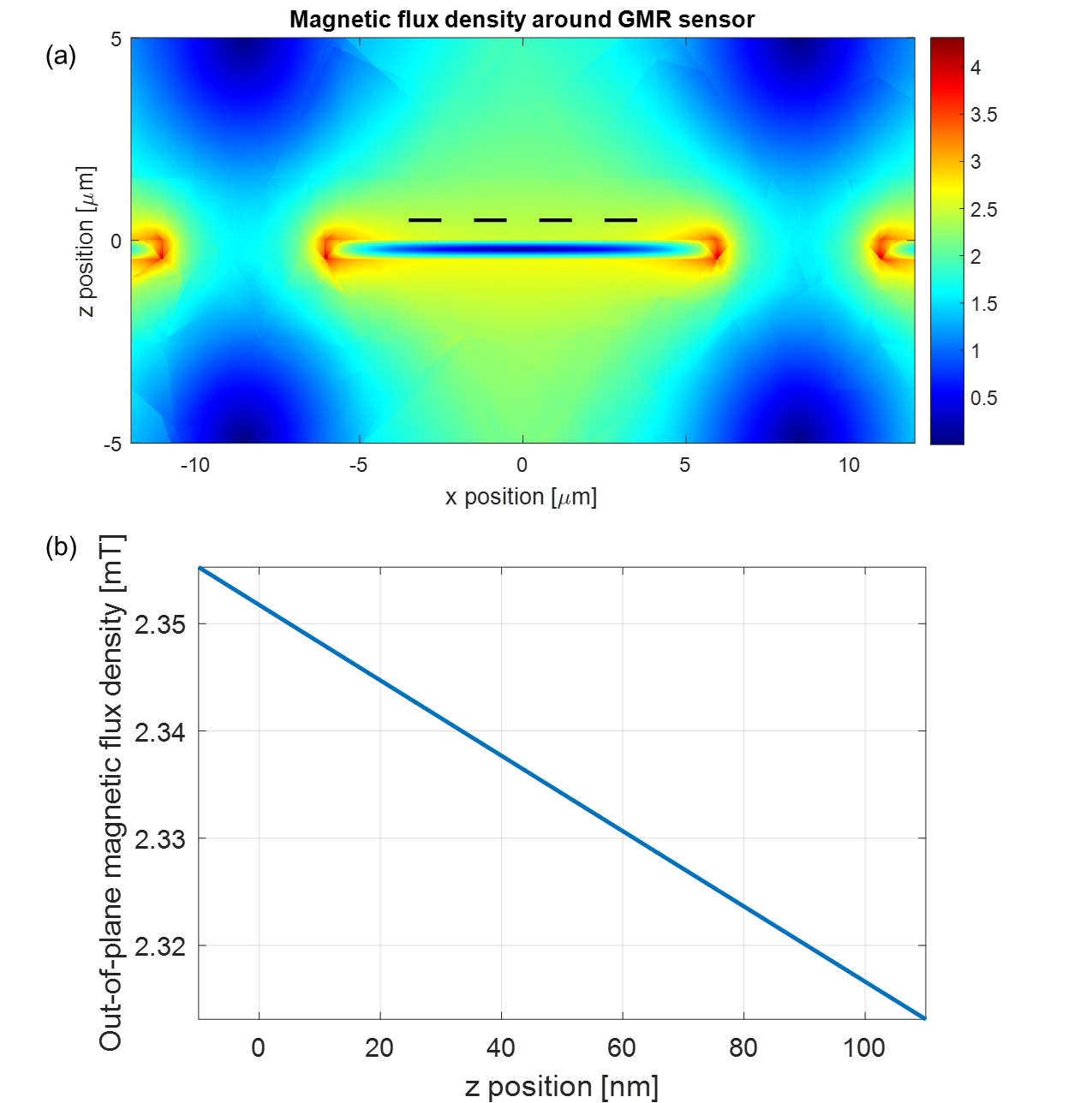


**Fig. S1.** (a) Finite element simulation of magnetic flux density distribution on xz plane around the field wire and sensor strips. Four 1 µm wide sensor strips are shown in black, located 500 nm above the field wire. The fabrication process is illustrated in Fig. S4. (b) Calculated magnetic flux density dependence on the vertical distance. The flux density decreases by 1.4% when the vertical distance increases from 0nm to 100 nm.


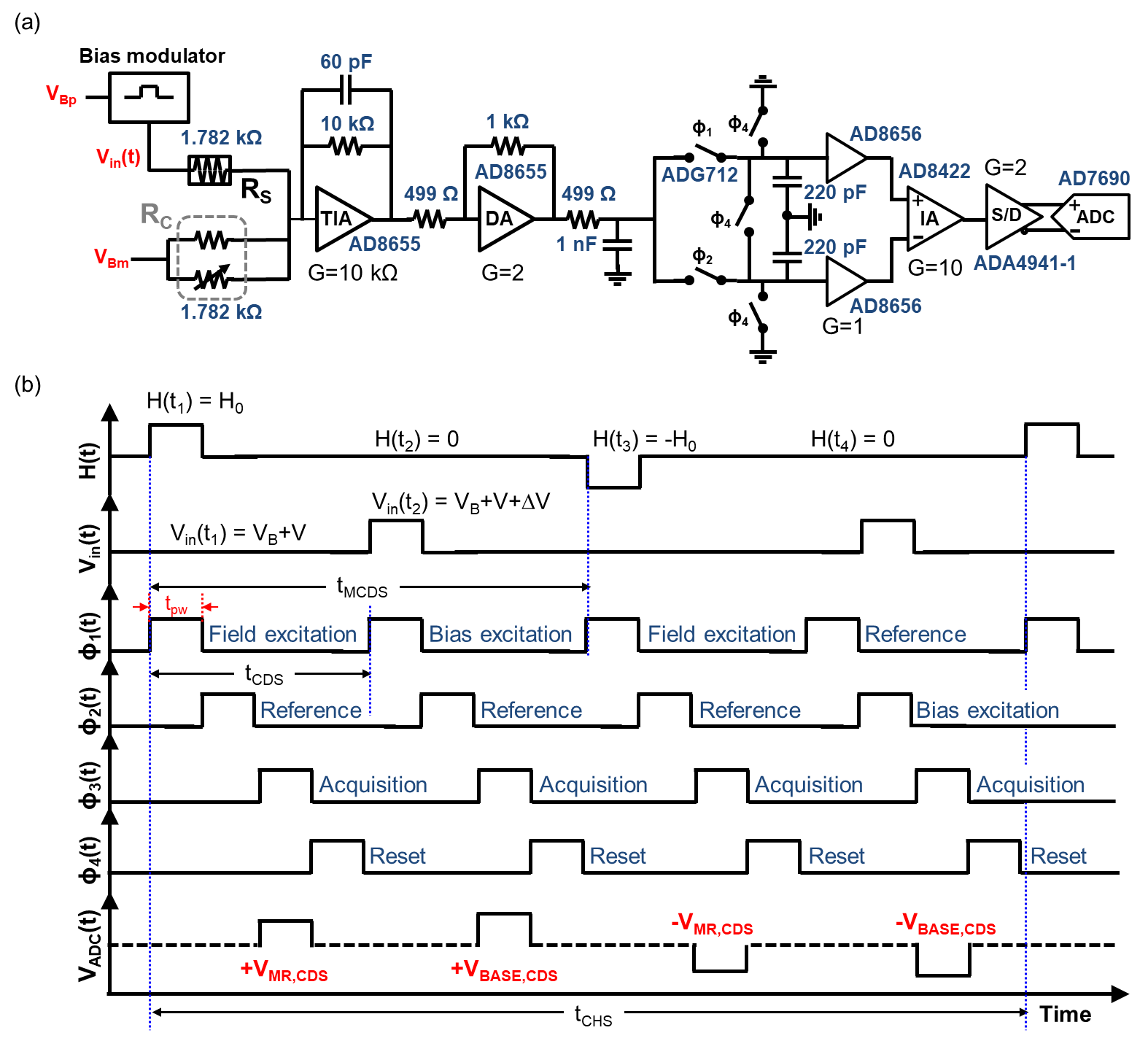


Fig. S2. (a) Detailed schematic of prototype circuit implementing MCDS. Global chopper stabilization is implemented with the bipolar magnetic field driver shown in Fig. S5. (b) Timing diagram of the CDS switches as well as the input voltage bias and excitation magnetic field. Four switching periods, Φ_1_ to Φ_4_ form one CDS cycle (t_CDS_). Two CDS operations are required to perform one MCDS (t_MCDS_), and one global chopper stabilization period (t_CHS_) has two CDS cycles.

Table S1. Summary of signal amplitudes at various nodes along the signal path.

|  | *t*_1_ (Φ_1_) | *t*_2_ (Φ_2_) | *t*_2_ (Φ_4_) | *t*_3_ (Φ_1_) | *t*_4_ (Φ_2_) | *t*_4_ (Φ_4_) |
| --- | --- | --- | --- | --- | --- | --- |
| *H*_B_(t) | *H*_0_ | 0 | 0 | -*H*_0_ | 0 | 0 |
| *R*_S_(t) | *R*_S_+Δ*R* | *R*_S_ | *R*_S_ | *R*_S_-Δ*R* | *R*_S_ | *R*_S_ |
| *V*_B_(t) | *V*_CM_ | *V*_CM_ | *V*_CM_ | *V*_CM_ | *V*_CM_ | *V*_CM_ |
| *I*_ΔR_(t) | I_ΔR_ | 0 | 0 | -I_ΔR_ | 0 | 0 |
| *V*_X+_ | *g*_1_ *I*_ΔR_+*V*_µ_+*V*_γ_ | *g*_1_ *I*_ΔR_+*V*_µ_+*V*_γ_ | 0 | - *g*_1_ *I*_ΔR_+*V*_µ_+*V*_γ_ | - *g*_1_ *I*_ΔR_+*V*_µ_+*V*_γ_ | 0 |
| *V*_X_­_-_ |  | *V*_µ_ | 0 |  | *V*_µ_ | 0 |
| *V*_X+_-*V*_X-_ |  | *g*_1_ *I*_ΔR_+*V*_γ_ | 0 |  | - *g*_1_ *I*_ΔR_+*V*_γ_ | 0 |
| *V*_ADC_ |  | *g*_1_*g*_2_*I*_ΔR_+(*g*_1_*V*_µ_+*V*_Σ_) |  |  | - *g*_1_*g*_2_*I*_ΔR_+(*g*_1_*V*_µ_+*V*_Σ_) |  |
| *V*_OUT_ |  | *g*_1_*g*_2_*I*_ΔR_+(*g*_1_*V*_µ_+*V*_Σ_) |  |  | *g*_1_*g*_2_*I*_ΔR_-(*g*_1_*V*_µ_+*V*_Σ_) |  |

In Fig. S2 and the simplified circuit diagram shown in Fig. 3, the 1/*f* noise and offset voltage, *V*_µ_, of the first stage are canclled by the MCDS operation. The noise component *V*_ɣ_ at input of the IA contains the mismatch of the CDS capacitors and the noise from the buffers and IA whereas *V*_ɛ_ accounts for noise from the IA, S/D, and ADC. Note that the ADC quantization noise is usually small enough that it can be ignored. The output sequence after the digital modulator is a repeating sequence of *V*_∆R_ *+ g*_2_*V*_ɣ_ *+ V*_ɛ_, *V*_∆R_ – (*g*_2_*V*_ɣ_ *+ V*_ɛ_), where *V*_∆R_ is *g*_1_*g*_2_*I*_∆R_. Because the signal of interest, *V*_∆R_­, and the noise component *g*_2_*V*_ɣ_ *+ V*_ɛ_ are separated in the frequency domain, digital averaging of the sequence (i.e., low pass filtering) fully suppresses the noise component leaving only *V*_∆R_. Compared with a conventional readout system utilizing spectral analysis, this method does not suffer from the additional post-processing time overhead due to the FFT.


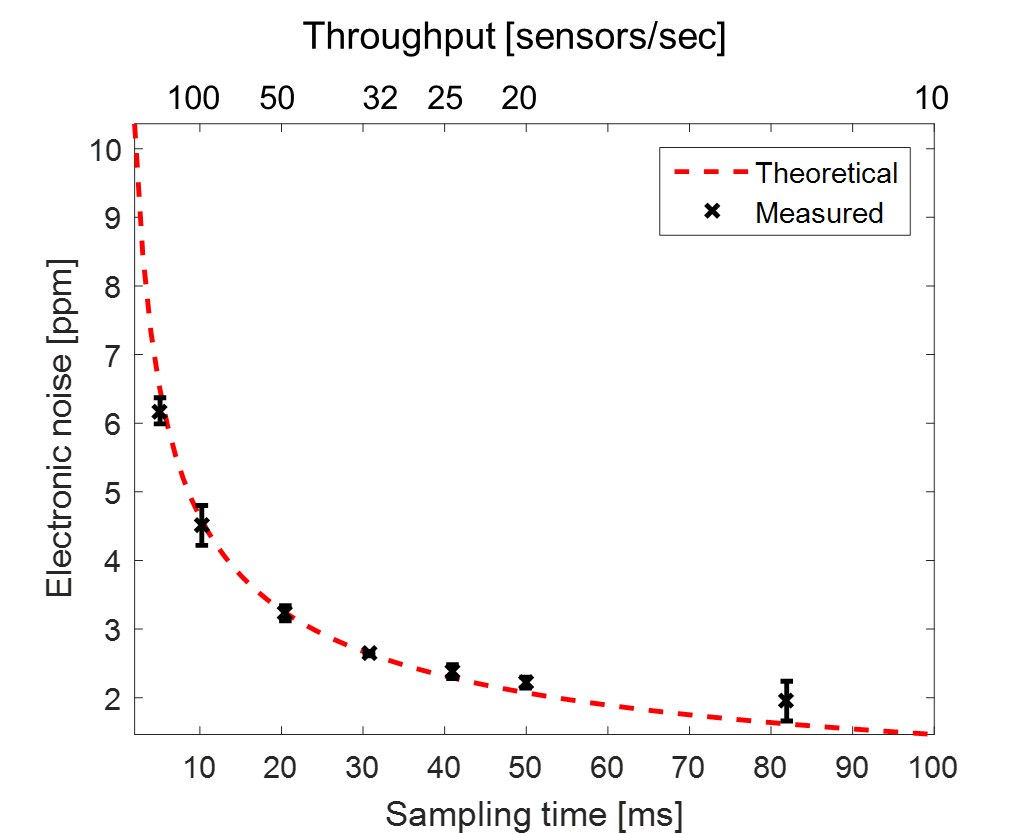


**Fig. S3.** Readout speed vs. electronic noise curve. The magnitude of operating in-plane magnetic flux density is 2.3 mT and the sensitivity of the GMR sensor to be measured is 5.2 Ω/mT at that magnitude. The red dashed curve shows the theoretical electronic noise versus sampling time, derived from the thermal noise analysis of the electronic circuits in Fig. S2(a). The measurement results using the prototype GMR SV sensor chip is overlaid. As a sampling time for a set of data sequence to draw one measurement point in a binding curve gets smaller, electronic noise gets increased. The throughput indicates the number of sensors to be read out per second.

Fig. S4. Fabrication process of GMR SV sensors with integrated, buried strip lines. The photolithography process steps are omitted for brevity.

a) 1 μm thick wet oxidation on silicon wafer.

b) Photolithography then patterned 500 nm deep field wires with photoresist preserved.

c) Fill patterns with a 5 nm Ti / 490 nm Au / 5 nm Ti metal stack followed by a lift-off by soaking samples in acetone.

d) Deposit 500 nm thick SiO_2_ at 350 °C. Then apply blanket ion milling for a short period of time to remove possible rabbit ears on the surface of the SiO_2_ layer.

e) Deposit GMR SV film stack.

f) Photolithography then pattern sensor strips using ion milling.

g) Photolithography then deposit 5 nm Ti / 490 nm Au / 5 nm Ti metal layer for making interconnection leads followed by a lift-off.

h) Photolithography then deposit 15 nm SiO_2_ / 15 nm Si_3_N_4_ / 15 nm SiO_2_ passivation layer to protect the GMR SV sensors. Next perform a lift-off to pattern the thin passivation layer.

i) Photolithography then deposit 150 nm SiO_2_ / 150 nm Si_3_N_4_ / 150 nm SiO_2_ passivation layer to protect GMR SV sensors.

**Fig. S5.** Design of the bipolar magnetic field driver. The on-chip magnetic field wires are modeled as a network of resistors, capacitors and an inductor. Switching the polarity of the current changes the direction of the magnetic field. An amplifier and an ADC monitor the coil current and regulate the value of *R*_DAC_ to guarantee a constant *I*_coil_.

**Fig. S6.** Block diagram of the data acquisition circuits. AFE/ADC implements the proposed MCDS and global chopper stabilization techniques. The microprocessor (ARM32) communicates with an external PC via USB using the Test and Measurement Class protocol for fast control and data transmission. A 9V battery is used for system power.

**Fig. S7.** Photograph of the proposed system where (a) shows the prototyped data acquisition system on PCB with different sections labeled and (b) shows a zoomed-in view of the fabricated sensor chip with a reaction well attached.
